# The Effect of Multiple Single Nucleotide Polymorphisms in the Folic Acid Pathway Genes on Homocysteine Metabolism

**DOI:** 10.1155/2014/560183

**Published:** 2014-01-12

**Authors:** Shuang Liang, Yuanpeng Zhou, Huijun Wang, Yanyan Qian, Duan Ma, Weidong Tian, Vishwani Persaud-Sharma, Chen Yu, Yunyun Ren, Shufeng Zhou, Xiaotian Li

**Affiliations:** ^1^Obstetrics and Gynecology Hospital, Fudan University, Shanghai 200011, China; ^2^Department of Obstetrics and Gynecology, Shanghai Medical College, Fudan University, Shanghai 200032, China; ^3^Shanghai Key Laboratory of Female Reproductive Endocrine Related Diseases, Shanghai 200011, China; ^4^State Key Laboratory of Genetic Engineering, Institute of Biostatistics, School of Life Science, Fudan University, Shanghai 200433, China; ^5^Children's Hospital of Fudan University, Shanghai 201102, China; ^6^Institute of Biomedical Sciences, Fudan University, Shanghai 201508, China; ^7^Department of Pharmaceutical Sciences, College of Pharmacy, University of South Florida, Tampa, FL 33612, USA; ^8^Central Lab, Shanghai Xuhui Central Hospital, Shanghai 200031, China

## Abstract

*Objective*. To investigate the joint effects of the single nucleotide polymorphisms (SNPs) of genes in the folic acid pathway on homocysteine (Hcy) metabolism. *Methods*. Four hundred women with normal pregnancies were enrolled in this study. SNPs were identified by MassARRAY. Serum folic acid and Hcy concentration were measured. Analysis of variance (ANOVA) and support vector machine (SVM) regressions were used to analyze the joint effects of SNPs on the Hcy level. *Results*. SNPs of MTHFR (rs1801133 and rs3733965) were significantly associated with maternal serum Hcy level. In the different genotypes of MTHFR (rs1801133), SNPs of RFC1 (rs1051266), TCN2 (rs9606756), BHMT (rs3733890), and CBS (rs234713 and rs2851391) were linked with the Hcy level adjusted for folic acid concentration. The integrated SNPs scores were significantly associated with the residual Hcy concentration (RHC) (*r* = 0.247). The Hcy level was significantly higher in the group with high SNP scores than that in other groups with SNP scores of less than 0.2 (*P* = 0.000). Moreover, this difference was even more significant in moderate and high levels of folic acid. *Conclusion*. SNPs of genes in the folic acid pathway possibly affect the Hcy metabolism in the presence of moderate and high levels of folic acid.

## 1. Introduction

The folic acid pathway is essential for hundreds of intracellular transmethylation reactions including DNA methylation and DNA synthesis, processes that are closely related to homocysteine (Hcy) metabolism [1, 2]. Folic acid deficiency and abnormal metabolism of folic acid and Hcy not only play an important role in neural tube defects (NTDs) [3], but also are key factors for congenital heart disease, cleft lip and palate, late pregnancy complications, premature labor different kinds of neurodegenerative and psychiatric diseases, and cancer [3–10]. It was reported that folic acid preconceptional supplementation is effective for NTD prevention. However, it remains unclear whether 30–50% of cases are still unpreventable with folic acid supplementation [2].

Variations in genes that play key roles in the folic acid cycle have been widely investigated, where single nucleotide polymorphisms (SNPs) have been found to be associated with folic acid and Hcy metabolism. The variants of MTHFR and RFC1 were found to interact with Hcy levels [11, 12], while the combined effects of the 2 MTHFR polymorphisms (rs1801133 and rs1801131) were found to be associated with Hcy concentration [13]. However, studies have been limited to one or a few SNPs with joint effects [11, 14–16].

With the rapid development of ongoing high-throughput human gene sequencing and bioinformatics, abundant SNPs can be identified. Support vector machines (SVMs) are a classic supervised machine learning algorithm typically used for classification and regression analysis. SVM has been widely used in solving biological problems, including gene function prediction, gene expression data analysis, and even cancer diagnosis [12, 17, 18]. It has also been applied to the analysis of SNPs.

The aim of the study is to identify the genotypes of 18 SNPs using MassARRAY and to investigate an association between the cumulative effects of 18 SNPs in the 9 genes of the folic acid pathway and Hcy metabolism.

## 2. Material and Method 

### 2.1. Study Population

Four hundred pregnant women at 11–25 gestational weeks were enrolled in the study conducted at the Obstetrics and Gynecology Hospital of Fudan University, China, from April to May 2011. Women included in the study were not smokers, did not drink alcohol, had no chronic diseases, and were not taking prescription medications. Blood samples were taken from fasting subjects. The serum was separated for the measurement of the concentrations of folic acid and Hcy and the remaining blood clots were used for DNA extraction and genotyping. All individuals enrolled in this study signed the informed consent and the study was approved by the Ethics Committee of the Obstetrics and Gynecology hospital of Fudan University, China.

### 2.2. Biochemical Measurement of Serum Folic Acid and Hcy Concentrations

Serum folic acid concentration was measured by a chemiluminescent microparticle immunoassay (Architect Folic acid Reagent; Abbott, Lisnamuck, Longford) using the ARCHITECT I Systems following the manufacturer's recommended protocols.

Serum Hcy measurements were carried out by Liquid Chromatography Coupled to Tandem Mass Spectrometry (LC/MS/MS) using an API 3000 LC/MS/MS system (Applied Biosystem) equipped with an electrospray ionization interface that was used in the positive ion mode ([M+H]^+^) according to the manufacturer's instructions.

### 2.3. Identification of SNPs Using MassARRAY

**Figure 1:**
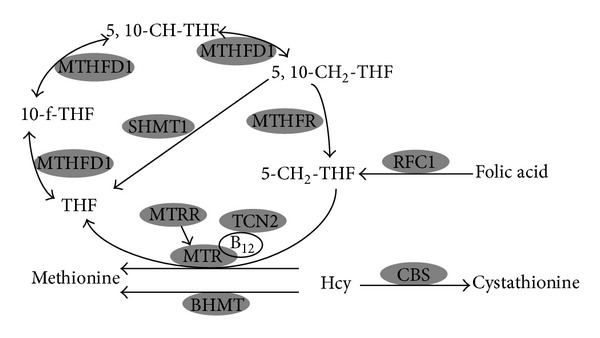
Folic acid gene and homocysteine gene pathways. Hcy: homocysteine; BHMT: betaine Hcy methyltransferase; CBS: cystathionine beta synthase; MTHFD1: methylenetetrahydrofolate dehydrogenase1; MTHFR: methylenetetrahydrofolate reductase; MTR: methionine synthase; MTRR: methionine synthase reductase; RFC1: reduced folate carrier 1; TCN2: transcobalamin2; SHMT1: serine hydroxymethyltransferase1.

18 SNPs in 9 folic acid pathway (Figure 1) related genes that have been documented in the literature to be associated with Hcy related disease were selected in our population [19–22] based on the CHB data using the following criteria: MAF > 0.1 by the Haploview program (version 4.0) (Table 1).

Genomic DNA was prepared from peripheral leukocytes using Relax Gene blood DNA System (Relax Gene; TIANGEN, Beijing, China). The genotypes were determined by the Sequenom MassARRAY MALDI-TOF system. Primer sequences of the 18 SNPs were shown in Supplementary Table 1 (see Supplementary Table 1 in the Supplementary Material available online at http://dx.doi.org/10.1155/2014/560183).

### 2.4. SVM Regression Model

SVM regression model was used to analyze the relationship between the SNPs of genes in the folic acid pathway and the changes in serum Hcy concentration. To minimize the effects of folic acid concentration on Hcy concentration, the residual homocysteine concentration (RHC, RHC = actual Hcy concentration − predicted value with a liner regression function of folic acid) was used as the dependent variable. The predicted RHC was defined as the SNP scores. For the independent variables (features) of the SVM regression model, we coded each SNP as two independent variables. For example, if a SNP has three types, AA, Aa, and aa, its coding features would be only two independent variables, which are feature_AA and feature_Aa, following Table 2. Thus, the input space initially has 30 independent variables for 15 SNPs.

Basically, the SVM regression model was implemented using “SMOReg” algorithm of Weka software package with default parameters, where C equals 1.0; the kernel is polynomial kernel with exponent value equals 1.0. Using all of the 15 SNPs to construct a data model would result in suboptimal accuracy because the different variables may contain overlapping information that disturbs the model-constructing process and thus variable selection was needed. Variable selection out of the total 15 SNP variables was conducted using recursive addition and stepwise addition of the input variables. The basic idea of the method was that, beginning with two features, we tried to add features to the SVM regression model which would improve the correlation coefficient most and then add another feature until none of the added features could improve the model or the improvement was less than the 0.01 correlation coefficient (see van Looy et al.'s paper [23] for more details).

After features selection, a tenfold cross-validation model was used to assess the SVM regression model. In this method, we divided the dataset into 10 subsets of approximately equal size and built the model ten times, each time leaving out one of the subsets as testing set and the others as training sets.

### 2.5. Statistical Analysis

Statistical analyses were performed using SPSS (SPSS Inc., Chicago, IL, USA), version 16.0 for Windows. A *P* value of <0.05 was considered statistically significant. The Hardy-Weinberg equilibrium constant was assessed using the chi-squared (*χ* ^2^) test. Pairwise linkage disequilibrium of SNPs was estimated using Haploview. The square of the correlation coefficient (*r* ^2^) between markers was used to define linkage using the data from the study population. Linear regression was used for detecting the association between folic acid and Hcy concentration, while analysis of variance (ANOVA) was used for the association analysis between SNP and Hcy concentration with serum Hcy concentration as dependent variable, serum SNP genotypes as the fix factor, and folic acid concentration as covariance.

## 3. Results

### 3.1. Demographic Characteristics of the Participants

Genotyping results were available for 386 of the 400 subjects (96.5%). A total of 14 subjects were excluded: 5 samples due to sample hemolysis and 9 samples due to sample DNA degradation. Data on serum folic acid and Hcy were available for all subjects. The regression equation used was

(1) Hcy concentration (umol/L)  =−0.094×folic acid concentration (ng/mL)   +6.624

(*r* = −0.385, *P* < 0.01, Supplementary Figure 1).

All subjects were genotyped for 18 SNPs. Three SNPs were eliminated from further analysis: TCN2 (rs1801198) showed significant (*P* < 0.05) deviation from the Hardy-Weinberg proportions and CBS (rs5742905) and MTR (rs74767314) were monomorphic. The remaining 15 SNPs are shown in Table 1. The genotyping call rate for each SNP ranged from 96% to 100%. There was no evidence for linkage disequilibrium in our database (*P* > 0.05, Supplementary Figure 2). There was no significant difference in age, gestational weeks, parities, and pregnancies among each genotype of the 15 SNPs (*P* > 0.05, Supplementary Tables 2–5).

### 3.2. The Effects of SNPs on Serum Hcy Concentration Adjusted for Folic Acid Concentration

MTHFR SNPs (rs1801133 and rs3737965) were associated with serum Hcy concentrations which were adjusted for folic acid concentration (Table 3). There were only two cases with homozygous MTHFR SNP (rs3737965). Due to the low frequency of variants for MTHFR SNP (rs3737965) polymorphism, it was not included in the data analysis.

**Figure 2:**
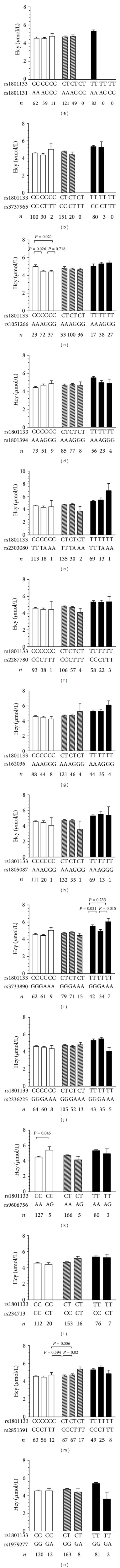
The Relationship between Hcy Concentration and SNPs Based on the Different Genotypes of MTHFR (rs1801133). Hcy: Homocysteine.

Figure 2 shows the effects of SNPs on Hcy concentration in the different genotypes of MTHFR (rs1801133) after the Hcy concentration was adjusted for folic acid concentration. The SNPs MTHFR (rs1801133) CC, RFC1 (rs1051266), and TCN2 (rs9606756) were significantly associated with Hcy concentration (Figures 2(c) and 2(k)). A similar association was observed with the SNPs CBS (rs2851391) in MTHFR (rs1801133) CT and MTHFR (rs3733890) and CBS (rs234713) in MTHFR (rs1801133) TT (Figures 2(m), 2(i), and 2(n)).

### 3.3. SVM Model of Multiple SNPs and the Residual Hcy Concentration

**Figure 3:**
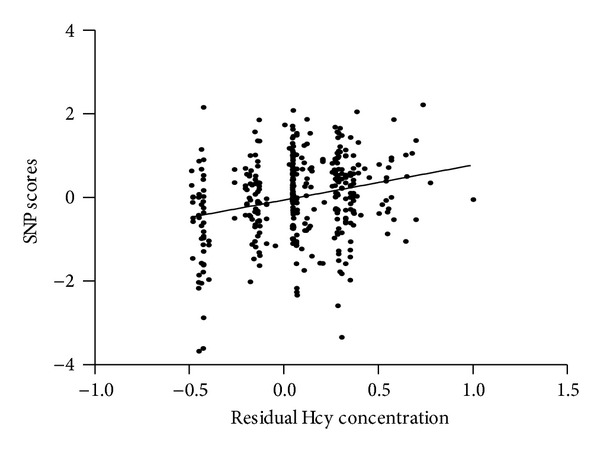
The relationship between SNP scores and residual Hcy concentration. Hcy: homocysteine.

In the SVM regression, five SNPs were selected: MTHFR (rs1801133, rs1801131, and rs3737965), CBS (rs234713), and BHMT (rs3733890). The weights of the five SNP variables are shown in Table 4 and the relationship between SNP scores and residual Hcy concentration is shown in Figure 3. The correlation coefficient between RHC and SNP scores was 0.275 in training sets and only 0.247 in the cross-validation combined test sets (Supplementary Table 6).

**Figure 4:**
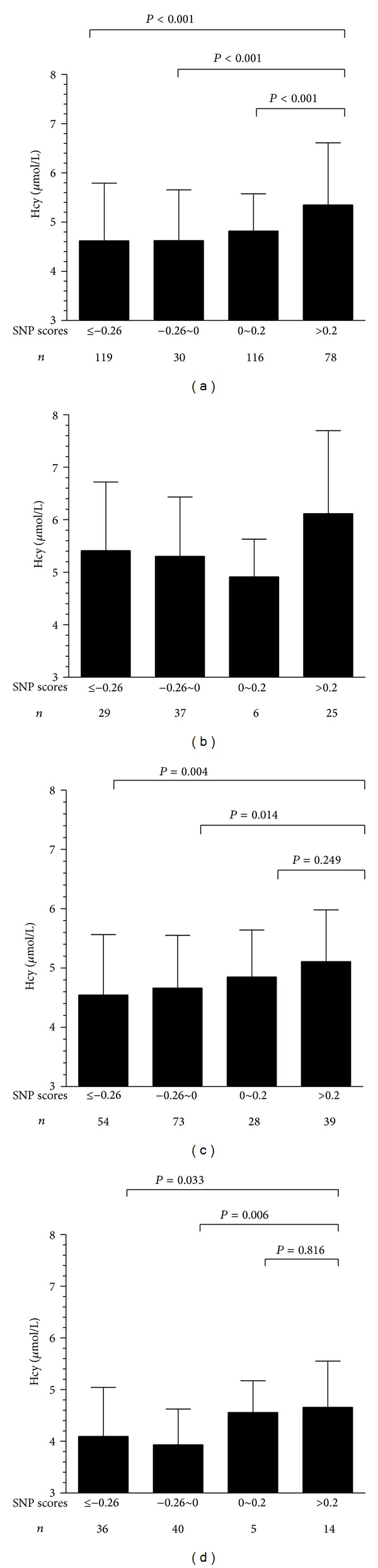
Changes in Hcy among the different groups of SNP scores. (a) In all subjects, (b) low folic acid (less than 13.1 ng/mL (25%)), (c) moderate folic acid concentration (between 13.1 ng/mL (25%) and 18.4 ng/mL (75%)), and (d) high folic acid concentration (more than 18.4 ng/mL (75%).

All subjects were divided into four groups according to the 25%, 50% and 75% of the SNP scores (−0.26, 0, and 0.2, resp.). The Hcy concentration was significantly higher in the group with SNP scores of more than 0.2 than that in groups with SNP scores less than 0.2 (*P* < 0.01, Figure 4(a)). For those with folic acid levels more than 25% (13.1 ng/mL), a possible interaction between Hcy concentration and SNP scores was detected (*P* < 0.05) (Figures 4(c) and 4(d)). However, for those subjects with folic acid levels less than 25%, SNP scores appeared not to be associated with Hcy concentration (Figure 4(b)).

## 4. Discussion

We first used SVM regression to predict Hcy concentration from the SNPs of genes in the folic acid pathway after analysis of the joint effect between MTHFR (rs1801133) and other genes related to Hcy metabolism. The results revealed that the integrate SNPs scores of SVM were significantly associated with Hcy concentration, especially at moderate and high levels of folic acid. This finding suggests that the variations in the genes of the folic acid pathway may be an important contributor to Hcy related diseases in women with moderate and high folic acid levels from folic acid supplementation.

Our finding that the SNPs in MTHFR (rs1801133, rs1801131, and rs3737965), RFC1 (rs1051266), CBS (rs2851391 and rs234713), TCN2 (rs9606756), and BHMT (rs3733890) were associated with the Hcy level adjusted for folic acid level is partly consistent with previous studies. Moreover, it is worth noting that the variation of MHTFR (rs1801133) was included, along with other SNPs. The thermolabile protein MTHFR is of great importance for the regulation of available 5-MTHF, which serves as the main circulating folate necessary for Hcy remethylation. MTHFR (rs1801133) can result in 50–60% reduced enzyme activity, while MTHFR (rs1801131) can also decrease MTHFR activity [24, 25]. MTHFR (rs3737965) was moderately associated with plasma folic acid concentration by genome-wide association studies [26]. RFC1 is necessary for the uptake of folic acids such as 5-MTHF and RFC1 (rs1051266) was reported to have an impact on folic acid and Hcy concentrations [19, 22]. BHMT (rs3733890), TCN2 (rs9606756), and CBS (rs2851391 and rs234713) have been reported to be associated with Hcy related diseases [27–29].

High-throughput techniques have spawned a mass of complex biological data. However, analysis of these data creates a bottleneck seen in current studies [30].

In our study, the joint effects of SNPs generated nonlinear, noisy, and complex data sets that also contained a great deal of irrelevant information. Despite the effects of serum folic acid concentration, we have successfully presented a SVM regression model that can evaluate the RHC by five SNPs of genes in the folic acid pathway as inputs. This model translated the complex SNP patterns into a simple output of SNP scores which was significantly related to the changes in Hcy concentration adjusted by serum folic acid concentration, and it was found that 5 out of 15 SNPs were useful as inputs. The RHC was constructed to eliminate the effects of folic acid, for folic acid itself can affect Hcy level. It was suggested that the SVM model could be a potential algorithm for predicting Hcy related diseases.

Furthermore, we found that the Hcy concentration was significantly higher in the group with SNP scores of more than 0.2 than that in groups with SNP scores of less than 0.2, especially for those with folic acid level more than 25%. However, there were no changes in Hcy concentration detected for those subjects with folic acid levels less than 25%. Abnormal metabolism of Hcy is related to many diseases, such as congenital heart disease [5], cleft lip with or without cleft palate [4], and NTDs [6]. The causes of these diseases have not been identified under normal concentrations of folic acid although folic acid periconceptional supplementation can effectively prevent many diseases related to folic acid deficiency. Our study provides the evidence that the joint effects of SNPs in the folic acid pathway may play an important role in Hcy related diseases, especially under sufficient support of folic acid.

In conclusion, the joint effects of SNPs in genes that belong to the folic acid pathway can affect Hcy metabolism especially under normal and high levels of folic acid. Further research that includes a bigger sample size is needed to test this SVM model.

## Supplementary Material

The supplementary material includes the basic feather among different SNP Genotypes, primer sequence of 18 SNPs, the relationship between the SNP scores and residual Hcy, linear relationship between folic acid and Hcy concentration and the linkage disequilibrium among SNPs.

## Figures and Tables

**Table 1:** 9 Folic acid related genes and 16 SNPs.

SNP	Location	Chromosome site	Gene	Nucleotide and amino acid change		*P*	MAF
rs1801131	11777063	1p36.3	MTHFR	A→C	Glu→Ala	0.992	0.169
rs1801133	11778965	1p36.3	MTHFR	C→T	Ala→Val	0.051	0.437
rs3737965	11789038	1p36.3	MTHFR	G→A	—	0.938	0.074
rs1805087	235115123	1q43	MTR	A→G	Asp→Gly	0.737	0.097
rs162036	7938959	5p15.31	MTRR	A→G	Lys→Arg	0.543	0.196
rs1801394	7923973	5p15.31	MTRR	A→G	—	0.424	0.249
rs2287780	7941304	5p15.31	MTRR	C→T	Arg→Cys	0.233	0.174
rs2303080	7931424	5p15.31	MTRR	T→A	Ser→Thr	0.582	0.09
rs3733890	78457715	5q13.1-q15	BHMT	G→A	Arg→Gln	0.434	0.3
rs2236225	63978598	14q24	MTHFD1	G→A	Arg→Gln	0.94	0.257
rs1979277	18172821	17p11.2	SHMT1	C→T	Leu→Phe	0.245	0.056
rs1051266	45782222	21q22.3	RFC1	A→G	His→Arg	0.057	0.463
rs234713	3360960	21q22.3	CBS	G→A	—	0.564	0.028
rs2851391	43360473	21q22.3	CBS	C→T	—	0.222	0.289
rs9606756	29336860	22q12.2	TCN2	A→G	lle→Val	0.736	0.018

*P*: the *P* value of Hardy-Weinberg; MAF: minimum allele frequency; SNP: single nucleotide polymorphisms; BHMT: betaine homocysteine methyltransferase; CBS: cystathione beta synthase; MTHFD1: methylenetetrahydrofolate dehydrogenase1; MTHFR: methylenetetrahydrofolate reductase; MTR: methionine synthase; MTRR: methionine synthase reductase; RFC1: reduced folate carrier 1; TCN2: transcobalamin2; SHMT1: serine hydroxymethyltransferase1.

**Table 2:** Feature coding of SNPs.

	Feature_AA	Feature_Aa
AA	1	0
Aa	0	1
aa	0	0

**Table 3:** Association between SNPs and Hcy.

SNP	Wide type		Heterozygous		Homozygous		*P*
	*N*	Hcy level	*N*	Hcy level	*N*	Hcy level	
MTHFR (rs1801131)	266	4.836 ± 0.064	108	4.665 ± 0.101	11	4.824 ± 0.316	0.154
MTHFR (rs1801133)	132	4.594 ± 0.089	171	4.736 ± 0.078	83	5.23 ± 0.113	<0.001
MTHFR (rs3737965)	331	4.838 ± 0.058	53	4.449 ± 0.144	2	5.098 ± 0.739	0.029
MTR (rs1805087)	312	4.804 ± 0.06	68	4.751 ± 0.128	3	4.31 ± 0.609	0.42
MTRR (rs162036)	245	4.793 ± 0.054	125	4.785 ± 0.121	13	4.913 ± 0.376	0.562
MTRR (rs1801394)	214	4.817 ± 0.072	150	4.74 ± 0.086	21	4.824 ± 0.229	0.49
MTRR (rs2287780)	257	4.813 ± 0.066	117	4.751 ± 0.098	8	4.596 ± 0.372	0.565
MTRR (rs2303080)	317	4.788 ± 0.059	61	4.819 ± 0.135	4	4.742 ± 0.525	0.838
BHMT (rs3733890)	183	4.835 ± 0.078	166	4.707 ± 0.082	31	4.973 ± 0.189	0.262
MTHFD1 (rs2236225)	212	4.837 ± 0.072	147	4.767 ± 0.087	26	4.574 ± 0.206	0.229
SHMT1 (rs1979277)	341	4.875 ± 0.057	43	4.855 ± 0.161	0	—	0.68
RFC1 (rs1051266)	73	4.895 ± 0.123	210	4.753 ± 0.072	100	4.766 ± 0.105	0.227
CBS (rs234713)	364	4.81 ± 0.055	22	4.503 ± 0.224	0	—	0.183
CBS (rs2851391)	200	4.75 ± 0.074	149	4.755 ± 0.86	37	5.075 ± 0.172	0.083
TCN2 (rs9606756)	373	4.793 ± 0.054	13	4.785 ± 0.291	0	—	0.979

Values are presented as mean ± standard deviation.

*P*: the *P* value of analysis of variance between 3 different genotypes of SNPs and homocysteine concentration. The covariate folic acid concentrations were from 14.584 to 15.362 ng/mL. Hcy: homocysteine; SNP: single nucleotide polymorphisms.

**Table 4:** Weights of SNP variables in the SVM model.

SNP	Genotype	Weight
MTHFR (rs1801133)	TT	0.503
MTHFR (rs3737965)	CT	−0.414
CBS (rs234713)	AG	−0.319
BHMT (rs3733890)	AG	−0.264
MTHFR (rs1801131)	CA	0.196
Constant		−0.000

SNP: single nucleotide polymorphism; BHMT: betaine homocysteine methyltransferase; CBS: cystathione beta synthase; MTHFR: methylenetetrahydrofolate reductase.

## Abbreviations

ANOVA: Analysis of variance
BHMT: Betaine-homocysteine methyltransferase
CBS: Cystathionine *β* synthase
Hcy: Homocysteine
LC/MS/MS: Liquid Chromatography Coupled to Tandem Mass Spectrometry
LD: Linkage disequilibrium
MTHFD1: Methylenetetrahydrofolate dehydrogenase1
MTHFR: Methylenetetrahydrofolate reductase
MTR: Methionine synthase
MTRR: Methionine synthase reductase
NTDs: Neural tube defects
RFC1: Reduced folate carrier 1
RHC: Residual homocysteine concentration
SHMT1: Serine hydroxymethyltransferase1
SNPs: Single nucleotide polymorphisms
SPSS: Statistical packages for social sciences
SVM: Support vector machine
TCN2: Transcobalamin2.
