# Supplementary material for: The Effect of Multiple Single Nucleotide Polymorphisms in the Folic Acid Pathway Genes on Homocysteine Metabolism
Source: Biomed Res Int. 2014 Jan 12;2014:560183. doi: 10.1155/2014/560183 (PMC3913508; doi:10.1155/2014/560183)

Supplementary Table 1 Primer Sequence of 18 SNPs of Genes in the Folic Acid Pathway

SNP	forward primer	reverse primer	extend primer
rs9606756	ACGTTGGATGCCTGTGACCTCATTTGTACC	ACGTTGGATGCCCAACTTCTCTACCAGATG	cGCTGTCCATCTCTGGTA
rs2851391	ACGTTGGATGAGGATGGACCCTTCGGGAT	ACGTTGGATGGAATATCGAGGCATGTCCAG	ctCGCCCTCTGCAGATCA
rs3737965	ACGTTGGATGAGGAGCTGGTAAGAAGCCG	ACGTTGGATGACCCCCGGCAACGCCTCTCT	CGCCTCTCTCAGTCC
rs3733890	ACGTTGGATGTTGTTGCAGTCAGGAGTGTG	ACGTTGGATGAGTGAAGCTCATGAAGGAGG	ggggaGGCTTGGAGGCTGCCC
rs2287780	ACGTTGGATGGAATATTCCTGGTTTACCCC	ACGTTGGATGGAAAAACTTCCTTACCTGGC	CTTACCTGGCCAAGAG
rs162036	ACGTTGGATGGGAGCTGTGCAGTAAACAAG	ACGTTGGATGGGAGGAGATCCAACAAGCAG	aACAGGCATCTCGTACAAAGC
rs2236225	ACGTTGGATGCACTAACCTACAAACCCTTC	ACGTTGGATGATCGCACATGGCAATTCCTC	ttcaTCCATCATTGCAGACC
rs1979277	ACGTTGGATGGGAGGAGGTTGAGAGCTTC	ACGTTGGATGCTCCTTTAGAAGTCAGGCAG	cagaCAGGCAGAGGGAAGA
rs1805087	ACGTTGGATGCTTTGAGGAAATCATGGAAG	ACGTTGGATGTCTACCACTTACCTTGAGAG	gtcCCTTGAGAGACTCATAATGG
rs1801394	ACGTTGGATGGCAGAAAATCCATGTACCAC	ACGTTGGATGCTATATGCTACACAGCAGGG	AAGGCCATCGCAGAAGAAAT
rs1801198	ACGTTGGATGTTCCTCTCTCTCTTCCTCAC	ACGTTGGATGAGACATGCTGTTCCCAGTTC	cgCCCAGTTCTGCCCCA
rs1801131	ACGTTGGATGCTTGAAGGAGAAGGTGTCTG	ACGTTGGATGCTTCACAAAGCGGAAGAATG	TGCGTGATGATGAAATCG
rs1801133	ACGTTGGATGTGAAGAGCAAGTCCCCCAAG	ACGTTGGATGCCGAGAGGTAAAGAACGAAG	GAAGACTTCAAAGACACTT
rs1051266	ACGTTGGATGCGTAGAAGCAAAGGTAGCAC	ACGTTGGATGAGAAGCAGGTGCCCGTGGAA	gactAGCTCCGGTCCTGGCGGC
rs234713	ACGTTGGATGACAGAGAAAGCTGTGGGTG	ACGTTGGATGTTCCTGCCTCCCTCCGAACT	tgcaTCCCTCCGAACTCACTTG
rs2303080	ACGTTGGATGCACAGCATCAGGGCTGTTAC	ACGTTGGATGTAAAAGAGAGCACTGCGTCC	gtAAAATAAAGGCAGACACAA

Supplementary Table 2　Age Differences Among Different SNP Genotypes

	wide type		heterozygous		homozygous		P
	N	age	N	age	N	age	
rs1801133	132	28.18±3.77	170	28.50±3.33	83	29.23±3.96	0.118
rs1801131	265	28.50±3.60	108	28.69±3.76	11	27.91±3.45	0.756
rs1051266	73	29.22±3.56	209	28.31±3.45	100	28.61±4.00	0.178
rs1801394	213	28.64±3.66	150	28.35±3.76	21	28.76±2.30	0.725
rs1805087	311	28.55±3.66	68	28.60±3.45	3	31.67±4.5	0.334
rs2303080	316	28.50±3.55	61	28.98±4.15	4	426.25±0.96	0.285
rs2236225	212	28.55±3.71	146	28.50±3.57	26	28.96±3.48	0.837
rs1801198	340	28.58±3.74	43	28.42±2.75	0	28.56±3.64	0.945
rs2851391	198	28.82±3.99	148	28.26±3.21	37	28.35±3.31	0.542
rs3733890	183	28.81±4.00	165	28.30±3.16	31	28.45±3.91	0.422
rs2287780	256	28.53±3.54	117	28.81±3.88	8	26.5±1.20	0.206
rs162036	245	28.45±3.66	124	28.82±3.46	13	28.54±4.89	0.654
rs9606756	372	28.54±3.59	13	28.85±5.0	0	-	0.764
rs234713	363	28.59±3.69	22	27.91±2.67	0	-	0.397
rs3737965	330	28.55±3.61	53	28.45±3.87	2	31.5±3.54	0.510

Comparisons between different SNP genotypes and age using $\chi 2$ test

Values are presented as mean ±SD

Supplementary Table 3 The Differences in Gestational Weeks Among Different SNP Genotypes

	wide type		heterozygous		homozygous		P
	N	gestational weeks	N	gestational weeks	N	gestational weeks	
rs1801133	128	16.41±2.39	168	16.04±2.24	81	16.23±2.10	0.383
rs1801131	254	16.22±2.25	105	16.10±2.30	11	16.91±2.39	0.529
rs1051266	70	16.19±2.51	204	16.05±2.02	94	16.53±2.58	0.240
rs1801394	204	16.18±2.33	143	16.22±2.22	20	16.40±2.04	0.912
rs1805087	304	16.22±2.34	61	16.20±1.96	3	16.67±2.52	0.941
rs2303080	304	16.13±2.29	60	16.42±1.90	3	18.33±4.04	0.17
rs2236225	206	16.05±2.11	138	16.48±2.54	26	16.12±1.90	0.228
rs1801198	27	16.25±2.23	42	15.95±2.58	0	-	0.418
rs2851391	194	16.30±2.12	140	15.96±2.48	35	16.60±2.00	0.219
rs3733890	175	15.95±2.07	161	16.29±2.27	30	16.97±2.88	0.051
rs2287780	245	16.07±2.24	115	16.43±2.19	7	16.57±3.21	0.33
rs162036	235	16.37±2.25	121	15.91±2.13	12	16.58±3.70	0.193
rs9606756	359	16.21±2.26	12	16.33±2.57	0	-	0.852
rs234713	351	16.25±2.28	20	15.55±1.88	0	-	0.179
rs3737965	317	16.20±2.26	52	16.27±2.34	2	17.00±2.27	0.867

Supplementary Table 4 The Differences in Pregnancies among Different SNP Genotypes

	wide type		heterozygous		homozygous		P
	N	pregnancies	N	pregnancies	N	pregnancies	
rs1801133	131	1.57±0.94	170	1.58±0.83	83	1.76±1.04	0.268
rs1801131	264	1.63±0.96	108	1.58±0.82	11	1.55±0.69	0.866
rs1051266	73	1.70±0.89	209	1.62±0.97	99	1.54±0.80	0.505
rs1801394	213	1.60±0.87	149	1.62±0.96	21	1.76±1.14	0.726
rs1805087	312	1.57±0.87	66	1.85±1.11	3	1.33±0.58	0.172
rs2303080	315	1.63±0.94	61	1.56±0.83	4	1.00±0.00	0.260
rs2236225	212	1.59±0.84	145	1.66±1.01	26	1.62±1.02	0.874
rs1801198	339	1.61±0.92	43	1.65±0.90	0	-	0.575
rs2851391	199	1.64±0.90	147	1.56±0.93	36	1.67±0.81	0.730
rs3733890	182	1.67±0.94	165	1.56±0.93	31	1.58±0.77	0.544
rs2287780	255	1.66±0.99	117	1.56±0.77	8	1.12±0.35	0.230
rs162036	244	1.59±0.91	124	1.66±0.87	13	1.77±1.42	0.654
rs9606756	371	1.61±0.91	13	1.62±1.20	0	-	0.997
rs234713	364	1.61±0.90	22	1.70±1.26	0	-	0.669
rs3737965	329	1.62±0.93	53	1.62±0.181	2	1.00±0.00	0.636

Supplementary Table 5 The Differences in Parity Among Different SNP Genotypes

	wide type		heterozygous		homozygous		P
	N	parities	N	parities	N	parities	
rs1801133	131	0.07±0.25	170	0.09±0.28	83	0.13±0.34	0.284
rs1801131	264	0.09±0.29	108	0.09±0.29	11	0.00±0.00	0.567
rs1051266	73	0.11±0.32	209	0.10±0.30	99	0.07±026	0.658
rs1801394	213	0.08±0.28	149	0.10±0.30	21	0.10±0.30	0.870
rs1805087	312	0.08±0.28	66	0.14±0.35	3	0.00±0.00	0.344
rs2303080	315	0.10±0.29	61	0.08±0.28	4	0.00±0.00	0.773
rs2236225	212	0.09±0.29	145	0.08±0.28	26	0.12±0.33	0.2848
rs1801198	339	0.09±0.28	43	0.12±0.32	0	-	0.553
rs2851391	199	0.08±0.27	147	0.10±0.30	36	0.11±0.32	0.614
rs3733890	182	0.09±0.29	165	0.08±0.28	31	0.13±0.34	0.739
rs2287780	255	0.10±0.30	117	0.09±0.28	8	0.00±0.00	0.614
rs162036	244	0.07±0.26	124	0.13±0.34	13	0.08±0.28	0.219
rs9606756	371	0.09±0.29	13	0.15±0.38	0	-	0.426
rs234713	364	0.09±0.29	20	0.10±0.31	0	-	0.888
rs3737965	329	0.09±0.28	53	0.11±0.32	2	0.00±0.00	0.762

Supplementary Table 6. Relationship Between the SNP Scores and Residual Hcy

effective valuables	training data<br>correlation coefficient	cross-validation<br>correlation coefficient
MTHFR(rs 1801133)	0.231	0.161
MTHFR (rs 1801133/rs3733965)	0.256	0.221
MTHFR (rs 1801133/rs3733965)/CBS(rs234713)	0.261	0.226
MTHFR (rs 1801133/rs3733965)/ CBS (rs234713)/ BHMT(rs3737890)	0.27	0.242
MTHFR (rs 1801133/rs3733965)/ CBS (rs234713)/ BHMT(rs3737890)/MTHFR(rs1801131)	0.275	0.247

Hcy : homocysteine; SNP: single nucleotide polymorphisms; SVM: support vector machine

Supplementary Figure 1. The Relationship Between Folic Acid and Hcy Concentration.

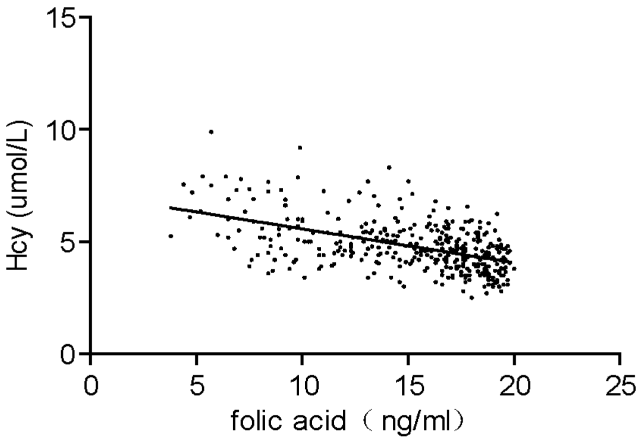

Supplementary Figure 2. Haploview LD Plots and Measures for Paired SNPs in the Genes
LD：Linkage Disequilibrium.
r^2 are indicated in the pair wise squares. No SNP was in the same block.

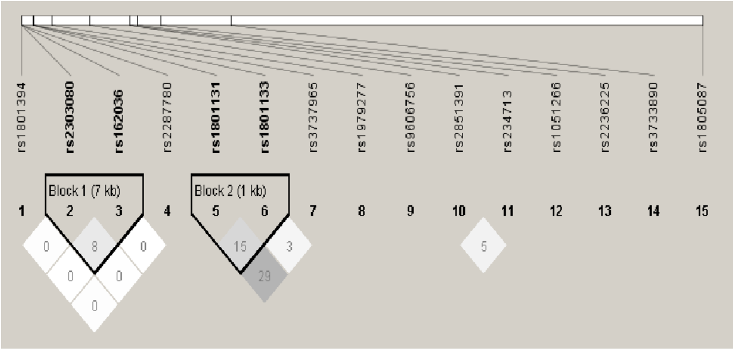

Supplement: Supplementary file 1 — The supplementary material includes the basic feather among different SNP Genotypes, primer sequence of 18 SNPs, the relationship between the SNP scores and residual Hcy, linear relationship between folic acid and Hcy concentration and the linkage disequilibrium among SNPs. [file 560183.f1.pdf]
